# Paging “Dr. Google”: Does Technology Fill the Gap Created by the Prenatal Care Visit Structure? Qualitative Focus Group Study With Pregnant Women 

**DOI:** 10.2196/jmir.3385

**Published:** 2014-06-03

**Authors:** Jennifer L Kraschnewski, Cynthia H Chuang, Erika S Poole, Tamara Peyton, Ian Blubaugh, Jaimey Pauli, Alyssa Feher, Madhu Reddy

**Affiliations:** ^1^Penn State University College of MedicineHershey, PAUnited States; ^2^The Pennsylvania State UniversityState College, PAUnited States; ^3^Family Health Council of Central PennsylvaniaCarlisle, PAUnited States

**Keywords:** qualitative research, prenatal care, pregnancy resources, Women, Infants, and Children Program, mhealth, mobile phones, smartphones, Internet, patient education, consumer health informatics

## Abstract

**Background:**

The prenatal care visit structure has changed little over the past century despite the rapid evolution of technology including Internet and mobile phones. Little is known about how pregnant women engage with technologies and the interface between these tools and medical care, especially for women of lower socioeconomic status.

**Objective:**

We sought to understand how women use technology during pregnancy through a qualitative study with women enrolled in the Women, Infants, and Children (WIC) program.

**Methods:**

We recruited pregnant women ages 18 and older who owned a smartphone, at a WIC clinic in central Pennsylvania. The focus group guide included questions about women’s current pregnancy, their sources of information, and whether they used technology for pregnancy-related information. Sessions were audiotaped and transcribed. Three members of the research team independently analyzed each transcript, using a thematic analysis approach. Themes related to the topics discussed were identified, for which there was full agreement.

**Results:**

Four focus groups were conducted with a total of 17 women. Three major themes emerged as follows. First, the prenatal visit structure is not patient-centered, with the first visit perceived as occurring too late and with too few visits early in pregnancy when women have the most questions for their prenatal care providers. Unfortunately, the educational materials women received during prenatal care were viewed as unhelpful. Second, women turn to technology (eg, Google, smartphone applications) to fill their knowledge gaps. Turning to technology was viewed to be a generational approach. Finally, women reported that technology, although frequently used, has limitations.

**Conclusions:**

The results of this qualitative research suggest that the current prenatal care visit structure is not patient-centered in that it does not allow women to seek advice when they want it most. A generational shift seems to have occurred, resulting in pregnant women in our study turning to the Internet and smartphones to fill this gap, which requires significant skills to navigate for useful information. Future steps may include developing interventions to help health care providers assist patients early in pregnancy to seek the information they want and to become better consumers of Internet-based pregnancy resources.

## Introduction

Little is known about how pregnant women are currently using Internet technologies (eg, websites, smartphone applications) and how these tools interface with medical care [1]. The Maternity Experiences Survey, a 2006-2007 national survey of postpartum women in Canada, found that women considered the most useful source of information during pregnancy to be their health care provider (32.2%), followed by books (22.3%), and personal experiences from a prior pregnancy (17.1%), although family and friends, the Internet, and prenatal classes were also noted [2]. Since that survey, the Internet and smartphones have become much more popular and, although patients report that physicians are their preferred source for information, the Internet is often the first resource accessed for health information because of its accessibility, wide availability, and low cost [3,4]. Women, in particular, are more likely to be health information seekers and to report that information obtained from the Internet helped them cope with their health conditions, including pregnancy [4,5]. Pregnant women who search the Internet for health information have a variety of resources from which to choose. One study investigating search terms for common obstetrical terms (eg, birth trauma, epidural, etc) found millions of websites, less than 4% of which were created or sponsored by physicians [6].

Given the wide availability of online resources for pregnant women, it is not surprising that nearly half (44%) of pregnant women are using these tools [1]. However, what remains less clear is the role technology has during pregnancy and the interaction between online resources and prenatal care. In this study, we sought to understand how women use technology during pregnancy through a qualitative study of pregnant women enrolled in the Women, Infants, and Children (WIC) program.

## Methods

### Setting and Participants

In spring 2013, we recruited pregnant women through posted advertisements at a Women, Infants, and Children (WIC) clinic in central Pennsylvania. The focus groups were conducted at the Cumberland/Perry Tapestry of Health (CP TOH) WIC clinic. The CP TOH provides the WIC program to over 3500 clients per month in both Cumberland and Perry counties in central Pennsylvania. Recruitment materials invited women to participate in a 90-minute focus group to discuss how smartphones may help women have healthier pregnancies. Interested women were screened in person at the clinic or by phone for eligibility. The eligibility criteria included being 18 years old or older, currently pregnant, and owning a smartphone. Women who met eligibility criteria and agreed to participate were scheduled for a focus group. Informed consent was obtained at the start of the focus group session. Each woman was compensated for her participation and childcare was provided. This study was approved by the Penn State University College of Medicine’s Institutional Review Board.

### Procedures

We conducted four focus groups, ranging between 2-6 participants each, with a total of 17 participants. The focus groups were conducted by 3 of the investigators (JLK, CHC, EP). The groups were held in a conference room at the WIC clinic. Prior to each session, participants completed a brief self-administered written questionnaire to assess demographic data (eg, race, ethnicity, weight, and height), adapted from the National Health and Nutrition Examination Survey, and smartphone use characteristics, adapted for our population from the Pew Research Internet Project [7].

The primary purpose of the focus groups was to determine participants’ requirements for a smartphone application aimed at helping them achieve a healthy pregnancy, while the current analysis reports on the subset of questions related to current technology use. Women were asked when they found out they were pregnant, when they called for prenatal care, and where they sought information prior to the first prenatal care visit. The focus group guide also included questions about the positive experiences and challenges they were having during their current pregnancy and how they had been using their smartphones and other sources of technology during the current pregnancy. Textbox 1 presents the questions from the interview guide analyzed in this manuscript. All sessions were audiotaped and transcribed. Members of the research team debriefed following each focus group session and focus groups were continued until it was agreed that thematic saturation was reached.

**Focus group interview guide.**

1. What’s been one of the most challenging things so far about this pregnancy?

     
a. Are these challenges your prenatal care providers have helped you with?

     
b. When did you have your first prenatal visit?

2. Tell us about something you’ve done to keep track of your pregnancy.

3. Do you talk about your pregnancy with others online (eg, on Facebook, Twitter, YouTube, or websites for expecting moms)?

4. You may or may not have seen them before, but there are a lot of smartphone applications that are about pregnancy. Have you downloaded any pregnancy applications?

     
a. What were your experiences with them?

### Data Analysis

Frequencies for data reported on the brief questionnaire are presented. Three members of the research team (JLK, IB, CHC) independently analyzed each transcript, using a thematic analysis approach. Data from the notes and transcripts were reviewed and codes were generated. Codes were then analyzed and categorized into over-arching themes independently by investigators and discussed, resulting in full agreement. Illustrative examples of the themes were selected and presented.

## Results

### Participant Characteristics

Table 1 displays the characteristics of the focus group participants. Women had an average age of 28 years and most were white. There were women representing all pregnancy trimesters. The vast majority reported using online social networking sites at least once a day (82%, 14/17).

**Table 1:** Participant characteristics (n=17).

Characteristics		n (%)
Age, years, median (range)		28 (21-38)
**Race**		
	White	14 (82)
	Other	3 (18)
**Parity**		
	Nulliparous	5 (29)
	Parous	12 (71)
**Number of weeks pregnant**		
	First trimester	7 (41)
	Second trimester	4 (24)
	Third trimester	6 (35)
**Pre-pregnancy weight category**		
	Normal weight	8 (47)
	Overweight	6 (35)
	Obese	3 (18)
**Smartphone operating system**		
	iOS	6 (35)
	Android	8 (47)
	Other	3 (18)
**Frequency of use of online social networking**		
	At least once a day	14 (82)
	At least once a week	3 (18)

### Themes

#### Overview

Technology use, either through Internet or smartphone applications, was the standard among our participants. Women sought the Internet and smartphone applications, in part because the prenatal care visit structure and how pregnancy-related information was delivered during prenatal care visits was not meeting their needs. Specifically, women reported using search engines (eg, Google) to answer pregnancy-related questions, which ranged from, “Am I pregnant?” to specific pregnancy-related symptoms (eg, abdominal pain, constipation, fatigue, heartburn). Women also frequently described using pregnancy-tracking smartphone applications (eg, BabyCenter) to follow their baby’s progress (eg, current size, organ development). Further, women used social media sites, including Facebook, to share their pregnancy experience and learn about the experiences of others (eg, videos of different birthing methods). The analysis of the women’s responses resulted in the identification of three overarching themes: (1) prenatal care structure is not patient-centered, (2) women used technology to fill gaps, and (3) technology has limitations in supporting their pregnancy-related needs.

#### Prenatal Care Structure Was Not Patient-Centered

In general, most women reported that the structure of their prenatal care did not meet their needs and thus was not patient-centered (ie, responsive to individual preference, needs, and values) in several key ways. First, the timing of the prenatal visits did not reflect when women wanted to see their provider. Multiple women commented that the first visit occurs too late and that there are too few visits early in pregnancy, when women had the most questions for their prenatal care providers:

They expected me to wait 13 weeks until I had a conversation with my doctor.

I know we can’t change the health care system…but on your first appointment, they say, “We’ll see you in 8 weeks…” and uh…That’s 2 months! What am I going to do?

Focus Group 1, Participant C; FG1, C

In contrast, women felt the clinic visit structure required too many visits toward the end of pregnancy. One woman suggested that the prenatal care visit structure was upside down:

I’m at 10 weeks, they don’t want to see me for another 6 weeks…they see you more at the end than at the beginning…they don’t have to see you so much at the end. [At the] beginning there are more questions about health and weight gain—that should be the priority.

FG1, D

In addition to the timing of clinic visits, women expressed that the supplemental information provided during these visits was not useful to them:

Today they gave me a whole bag of pamphlets and flyers and didn’t explain or go over them with me…and now I have to go home and try to go through them, while I have a kid running around…and when you’re a new mom, that’s overwhelming.

FG1, D

Another woman echoed this opinion:

They gave me pamphlets on my first appointment…here’s this one…here’s this one…and they bombard you with all this information.

FG2, B

Interestingly, the book of “What to Expect When Expecting”, previously considered a must-read for pregnant women, was unfamiliar to many of the women. Some women said they were given a copy of the book by their prenatal care provider but had not opened it because they found using a reference book to be outdated and not how they wanted to receive information. Those who had used the book expressed that much of the information it contained lacked usefulness:

[The book] says how to take care of your second child, well, I don’t need that—I’m a first time mother!

FG2, D

Another woman agreed,

Half that book doesn’t pertain to anyone or anything. Or it tells you about how you conceived the baby…I know this much already!

FG2, C

Another way in which women described how prenatal care lacked patient-centeredness was that the visits themselves felt structured around the clinics’ needs and not individualized toward the patients’ needs. For example, when discussing gestational weight gain:

It [the target weight to gain] wasn’t even my goal, it was theirs…They just gave me a paper. What good does that do at 28 weeks? I’m almost already there.

FG1, A

Another woman also commented on how her prenatal care felt routine and inconvenient:

I found out [I was pregnant] at 4 weeks…but I was new with this, so I was on it. They took me in for a blood test. That was it. They called to say, ‘Yes, congratulations’ and then called me at 9 weeks for a sonogram and a whole slew of blood work, and I asked specifically if I was going to see my doctor there and they said no. So I had to make a separate appointment following that, just to see my doctor, and pay for that visit.

FG1, C

The structure of the prenatal care led most of the participants to turn to Internet resources to provide them with pregnancy-related information.

#### Women Sought Technology to Fill Information Gaps and Share Their Pregnancy Experiences

Participants reported turning to technology to fill gaps largely attributed to limitations in their prenatal care, primarily the inability to ask their provider questions during their pregnancy. Most frequently, women reported using Google and smartphone applications (eg, Babycenter) as a means of finding information. One woman reported turning to Google to fill information gaps largely because of the prenatal visit limitations:

I did a lot of Google searches and Pinterest, my little mommy blog things, just looking at different things…my doctor didn’t tell me about round ligament pain, until, gosh, maybe 4 weeks ago…but I was having [the pain] from 13 weeks on.

FG1, C

Another woman echoed these thoughts:

I think they should see you at 6 [weeks pregnant]. Just because, with new moms, they’ll have all types of questions…they’re getting sick, feeling miserable. So they can get helpful tips, instead of getting all the info late...so women are going on Google to get their own answers because their doctors won’t see them.

FG1, D

Even when women had established care with their prenatal care provider, they almost all turned to Google first to answer their questions but would follow up with their provider if they still had concerns:

I’ll go on Google first. Then [if I don’t really understand], I’ll call my doctor.

FG3, C

Women also saw their use of technology during pregnancy as something unique to their generation.

Overall, Internet resources were used frequently and for a variety of pregnancy-related informational needs. When asked to recall a time when the Internet was used to look up things about pregnancy, one woman responded: “Every day!” [FG2, B]. More specific situations women reported included searching for different symptoms of pregnancy as well as baby-related information. These findings were similar across focus groups.

Some women believed that finding information online led them to read more, rather than less, about their pregnancy. For example, one woman reported:

I’m not a reader and I know this is going to sound really strange…I read more on my phone, tablet, or laptop than I would a book or magazine or pamphlet.

FG2, B

The Internet also offered resources in media formats typically not provided during prenatal care, yet desired by pregnant women. For example, some women reported watching videos of different types of births on the Internet. Another mother used videos of the developing baby to engage her family in the pregnancy: “I would show the kids the little video clips of the baby developing. Those are really good videos” [FG4, A]. In addition to filling information gaps for themselves, many of the women reported using technology as a way of connecting with their partners on the pregnancy, particularly for first-time dads:

Then I get the emails [from a pregnancy tracking website]…and I forward them to my husband, so that I feel like we’re both synced on what is going on.

FG1, D

Social media also provided opportunities for most women to learn about and share symptoms of pregnancy. Facebook, Instagram, and blogs were all mentioned as sources of obtaining information from others women who had similar experiences. Additionally, these venues were frequently reported as ways of sharing their personal pregnancy updates:

[Facebook plug-in program] automatically posts on your wall, so you don’t even have to worry about it…it does it for you, like every week, and it shows the progression of your baby.

FG1, D

However, many women reported being cautious about how much they shared on Facebook:

Some people share way too much…I put little things, like ‘20 week check…everything was doing really good’…I’m not friends with 18,000 people.

FG2, C

Overall, participants desired information, particularly early in their pregnancy prior to prenatal care and sought it independently through Google and other Internet applications.

#### Technology Had Limitations

Although all of the women in the focus groups reported using technology sources for pregnancy-related information, they did recognize significant limitations with this approach. For example, women reported needing to exercise caution when searching about symptoms they were having, to avoid receiving inaccurate information:

Sometimes you can Google something, like when I was having my round ligament pain for the first time…some things that came up were terrifying…you want to go to the ER right away! You definitely have to be careful and smart about your Google searches.

FG1, C

Women reporting using the Internet required multiple searches to find the specific information they were looking for:

I just add more keywords…or put my quotes in there…just a more specific, more refined search. And then it usually has a better answer. ’Cause sometimes when you’re just searching something, you just put in a very simple general description of what you want to search and it brings up all these different things.

FG1, C

Most women reported using a smartphone application to help keep track of their pregnancy progress, but that had significant limitations as well. For example, some women tracked their weight gain during pregnancy through a smartphone application, but when they found that they were gaining too much weight, there was no specific advice given to help women achieve weight gain goals:

The weight tracker I used…I stopped using it. It tells you where you should be [with weight gain], but it doesn’t make any sense. It says this is ‘you’ and this is where you should be, but what do I do [to get there]?

FG2, E]

Another woman echoed that simply tracking weight data without useful feedback on how to stay on track was unhelpful:

It’s like Google maps…saying you’re supposed to get there, but it doesn’t give you directions [on how to get there]

FG2, D

Other noted limitations included lacking a food diary to record what women eat.

Even though participants reported widespread use of technology sources for finding and sharing pregnancy-related information, they identified significant limitations leading some women to avoid using technology all together.

## Discussion

### Principal Findings

Our results suggest that women use the Internet and other sources of technology frequently during pregnancy in part because the current prenatal care visit structure does not allow women to seek advice when they want it the most, and thus is not patient-centered. Our findings echo those of a global study by Lagan and colleagues, who concluded that “the use of the Internet by pregnant women to seek health information and advice suggests a lack of information available from health professionals” [8]. National efforts to provide patient-centered care, described by the Institute of Medicine (IOM) as health care that is both respectful and responsive to a patient’s needs, preferences, and values, have begun in primary care with the creation of the Patient-Centered Medical Home [9]. As one of the most frequently used preventive health care services in the United States, prenatal care has not yet experienced a similar overhaul.

Given the lack of evidence on how prenatal care should ideally be delivered, the traditional model for prenatal care delivery in the United States has changed little over the last century and has been described as more “ritualistic than rational” [10-13]. The prenatal care visit structure typically starts with an initial prenatal visit (usually no earlier than 8 weeks of pregnancy), followed by infrequent visits until the third trimester. The more frequent visits toward the end of pregnancy support increased surveillance for signs of preeclampsia and preterm birth, which can change rather rapidly later in pregnancy. However, the importance of healthy behaviors during pregnancy is now better appreciated, especially in the presence of chronic disease (eg, diabetes, obesity, and hypertension) and detrimental health habits (eg, smoking alcohol use, nutritional) that require management as early as possible [14], preferably prior to conception.

Perhaps moving in the opposite direction, interventions have attempted to modify the current prenatal visit structure by actually reducing the number of visits [15,16]. A meta-analysis of seven trials with 60,724 women found that, despite no increase in adverse health outcomes, women were less satisfied with their care when the number of prenatal visits was reduced [10]. Further efforts to disseminate novel prenatal care approaches should demonstrate improved patient satisfaction as well as effectiveness as a challenger to the status quo, despite a lack of demonstrated effectiveness of traditional prenatal care [11]. One such effort is “CenteringPregnancy”, a group visit approach to prenatal care involving a care-provider physical examination (similar to the traditional appointment) followed by 90 minutes of a care provider-led educational group with women at similar pregnancy stages [17]. Although the prenatal care visit number is unchanged in CenteringPregnancy, the longer visits offer significant opportunity for information exchange and increased patient-centeredness and satisfaction.

Largely due to the lack of patient-centeredness in prenatal care, pregnant women in our study turned to technology for timely answers to their questions. This change appears to represent a generational shift compared to years prior, with women now using the Internet more and finding it to be more useful than family and friends [1]. Unlike their mothers, women in our study tended to ask “Dr. Google” first when investigating pregnancy questions, ranging from determining if they are pregnant, the etiologies of abdominal pain, and different birthing methods. Although most women utilized Internet resources, many commented on the challenges of finding reliable information that was helpful, instead of scary. Further, most women in our study used the Internet before contacting their prenatal care provider. Despite this extensive use of online resources, it is important that women still feel they can contact their provider for questions that remain.

Although technology use is widespread, online resources have significant limitations in meeting the informational needs for pregnant women. Importantly, there is a nearly complete lack of interaction between online resources and medical care. Kaimal and colleagues found in their study of online resources for obstetrics that fewer than 4% of websites were created and/or sponsored by physicians [6]. This suggests that the vast majority of online resources women utilize may be created in the absence of expert knowledge. As a result, significant skills are required by users to navigate the Internet for useful information. Internet-based tools may help improve access to pregnancy-related information; however, the benefit may be limited in certain populations due to lower rates of eHealth literacy, defined as “the ability to seek, find, understand, and appraise health information from electronic sources and apply the knowledge gained to addressing or solving a health problem” [18]. A recent study by Neter and Brainin found that eHealth literacy was higher among younger, more educated adults and that those with higher eHealth literacy were more likely to have positive outcomes from the information searched (ie, greater gains in health behaviors and positive interactions with their health care provider) [19]. Pregnant women tend to be younger, but those with lower socioeconomic status, such as the women in our study, may not derive equal benefit from these resources. Further research is necessary to determine how best to design technology to better serve this population.

However, this also presents a tremendous opportunity for medical systems to leverage the technology already available as well as further developing appropriate connections with medical expertise. For example, all of the women in our study used a pregnancy application to track their pregnancy. The limitations of these applications could easily be met by integrating evidence-based information from medical experts, such as including a discussion of the IOM’s guidelines for appropriate gestational weight gain instead of simply tracking weight gain.

Fortunately, women may be discussing questionable Internet information with their health care provider. Lagan and colleagues studied 303 midwives to assess their understanding of their patients’ use of the Internet and found that most (86%) had experienced a pregnant woman discussing information from the Internet with them in the past year [20]. Unfortunately, care providers may not be able to systematically evaluate such information: only 15% of midwives in Lagan’s study were aware of and able to describe indicators for quality online health information evaluation [20].

### Study Strengths and Limitations

Our study has several strengths. First, the role technology plays during pregnancy has been minimally explored in the literature, despite the growing use of technology. Additionally, to our knowledge, this is the only qualitative study investigating technology’s role in pregnancy for women enrolled in WIC, a health disparate population at greater risk for pregnancy complications. Our study also has limitations, including the recruitment of a convenience sample of volunteer pregnant women enrolled in WIC in Central Pennsylvania. As a result, volunteer bias is likely. Further, this population may be significantly different from women with higher socioeconomic status who experience fewer barriers to care access and may have different sources of information support. Therefore, our results may not be generalizable to pregnant women’s experiences in other settings. However, given the ubiquity of the Internet and largely standardized approach to prenatal care, we expect these results will be useful across the country. In addition, this study did not aim to compare technology with other information sources pregnant women use. Further qualitative and quantitative research on this topic is necessary in a large, more diverse group of women.

### Conclusions

Our results suggest several important next steps. Given how critical patient-provider communication is to the therapeutic relationship, the Internet should be considered by more providers as a forum for both dissemination of evidence-based education information and integration into the prenatal care structure. Importantly, none of the women in our study mentioned receiving online resources from their prenatal care provider, although it is established that providers are aware of and perhaps have even created such resources for their patients. Greater efforts are necessary to connect women with such resources, particularly early in pregnancy and prior to initiating prenatal care. For example, letting women know of reliable online resources when they first call in for an appointment to be scheduled weeks away may be useful. Further, as the role of technology is increasing in importance for pregnant women, understanding how best to leverage existing resources to facilitate healthy pregnancies will be necessary to meet women’s informational needs. Future steps may include developing interventions to help health care providers assist patients early in pregnancy seek the information they want and become better consumers of Internet-based pregnancy resources.

## Abbreviations

CP TOH: Cumberland/Perry Tapestry of Health
IOM: Institute of Medicine
WIC: Women, Infants, and Children

## References

[1] Grimes HA, Forster DA, Newton MS (2014). Sources of information used by women during pregnancy to meet their information needs. Midwifery.

[2] Public Health Agency of Canada (2009). What Mothers Say: The Canadian Maternity Experiences Survey.

[3] Hesse BW, Nelson DE, Kreps GL, Croyle RT, Arora NK, Rimer BK, Viswanath K (2005). Trust and sources of health information: the impact of the Internet and its implications for health care providers: findings from the first Health Information National Trends Survey. Arch Intern Med.

[4] Ybarra M, Suman M (2008). Reasons, assessments and actions taken: sex and age differences in uses of Internet health information. Health Educ Res.

[5] Shieh C, Broome ME, Stump TE (2010). Factors associated with health information-seeking in low-income pregnant women. Women Health.

[6] Kaimal AJ, Cheng YW, Bryant AS, Norton ME, Shaffer BL, Caughey AB (2008). Google obstetrics: who is educating our patients?. Am J Obstet Gynecol.

[7] Duggan M, Smith A (2013). Pew Research Internet Project.

[8] Lagan BM, Sinclair M, Kernohan WG (2011). What is the impact of the Internet on decision-making in pregnancy? A global study. Birth.

[9] Briere R, Institute of Medicine (2001). Crossing the Quality Chasm.

[10] Dowswell T, Carroli G, Duley L, Gates S, Gülmezoglu AM, Khan-Neelofur D, Piaggio GG (2010). Alternative versus standard packages of antenatal care for low-risk pregnancy. Cochrane Database Syst Rev.

[11] Novick G (2004). CenteringPregnancy and the current state of prenatal care. J Midwifery Womens Health.

[12] Alexander GR, Kotelchuck M (2001). Assessing the role and effectiveness of prenatal care: history, challenges, and directions for future research. Public Health Rep.

[13] Kirkham C, Harris S, Grzybowski S (2005). Evidence-based prenatal care: Part I. General prenatal care and counseling issues. Am Fam Physician.

[14] Johnson K, Posner SF, Biermann J, Cordero JF, Atrash HK, Parker CS, Boulet S, Curtis MG, CDC/ATSDR Preconception Care Work Group, Select Panel on Preconception Care (2006). Recommendations to improve preconception health and health care--United States. A report of the CDC/ATSDR Preconception Care Work Group and the Select Panel on Preconception Care. MMWR Recomm Rep.

[15] Sikorski J, Wilson J, Clement S, Das S, Smeeton N (1996). A randomised controlled trial comparing two schedules of antenatal visits: the antenatal care project. BMJ.

[16] McDuffie RS, Jr, Beck A, Bischoff K, Cross J, Orleans M (1996). Effect of frequency of prenatal care visits on perinatal outcome among low-risk women. A randomized controlled trial. JAMA.

[17] Shakespear K, Waite PJ, Gast J (2010). A comparison of health behaviors of women in centering pregnancy and traditional prenatal care. Matern Child Health J.

[18] Norman CD, Skinner HA (2006). eHealth Literacy: Essential Skills for Consumer Health in a Networked World. J Med Internet Res.

[19] Neter E, Brainin E (2012). eHealth literacy: extending the digital divide to the realm of health information. J Med Internet Res.

[20] Lagan BM, Sinclair M, Kernohan WG (2011). A Web-based survey of midwives' perceptions of women using the Internet in pregnancy: a global phenomenon. Midwifery.

